# Easy-to-Assembly System for Decellularization and Recellularization of Liver Grafts in a Bioreactor

**DOI:** 10.3390/mi14020449

**Published:** 2023-02-14

**Authors:** Maurício Felisberto Borges, Natasha Maurmann, Patricia Pranke

**Affiliations:** 1 Hematology and Stem Cell Laboratory, Faculty of Pharmacy, Universidade Federal do Rio Grande do Sul (UFRGS), Porto Alegre 90610-000, Brazil; 2 Postgraduate Program in Physiology, Universidade Federal do Rio Grande do Sul (UFRGS), Porto Alegre 90050-170, Brazil

**Keywords:** decellularized liver extracellular matrix, mesenchymal stromal/stem cells (MSCs), regenerative medicine, tissue engineering

## Abstract

Decellularization of organs creates an acellular scaffold, ideal for being repopulated by cells. In this work, a low-cost perfusion system was created to be used in the process of liver decellularization and as a bioreactor after recellularization. It consists of a glass chamber to house the organ coupled to a peristaltic pump to promote liquid flow through the organ vascular tree. The rats’ liver decellularization was made with a solution of sodium dodecyl sulfate. The recellularization was made with 10^8^ mesenchymal stromal/stem cells and cultivated for seven days. The decellularized matrices showed an absence of DNA while preserving the collagen and glycosaminoglycans quantities, confirming the efficiency of the process. The functional analyses showed a rise in lactate dehydrogenase levels occurring in the first days of the cultivation, suggesting that there is cell death in this period, which stabilized on the seventh day. Histological analysis showed conservation of the collagen web and some groups of cells next to the vessels. It was possible to establish a system for decellularization and a bioreactor to use for the recellularization method. It is easy to assemble, can be ready to use in little time and be easily sterilized.

## 1. Introduction

Liver diseases, from cirrhosis to hepatocellular carcinoma, are still a major cause for medical intervention in the world [1]. They account for approximately 3.5% of total deaths worldwide [2]. Today, the only viable treatment for hepatic failure is orthotopic liver transplantation [3], the liver being the second most transplanted solid organ, representing 23% of all solid organ transplantation procedures [4]. But there is an increasing deficit of donors, which results in the treatment of all patients becoming a constant challenge [5].

Tissue engineering, using the association of a variety of materials in association with biological factors, [6] has been able to offer viable options for treating several medical conditions, although with no apparent optimal solution. The solutions offered are still not fully developed and need more substantial evidence before their clinical application can be made.

Decellularization has been gaining space in the field of regenerative medicine due to its capacity for creating scaffolds that retain the original characteristics of the target organ, such as the microstructural architecture, the vascular tree and the biochemical composition of the extracellular matrix (ECM). The potential application of this technique has been demonstrated for entire organs such as the heart [7], kidney [8], liver [9], and several other types of tissue [10,11]. The method also offers the possibility of using organs that are unsuitable for transplant, such as in cases of cardiac death of the donor [9]. The ECM containing natural biomaterials resulting from decellularization is currently being used extensively as hydrogels to produce bioinks [12].

Various approaches have been successfully reported in the decellularization process, being divided into three main classes: biological, chemical and physical processes [6,13]. Biological processes consist of the use of enzymes such as proteases and nucleases. The main problem with this method is that the prolonged exposure can damage the tissue ultrastructure and remove collagen and glycosaminoglycans (GAGs) [6,13]. Physical processes consist of promoting rapid alterations in temperature or pressure to induce cell burst. Although this method better preserves the ECM structure, it is less efficient in removing the cellular material [13]. Chemical processes consist of using acids, bases and detergents to destroy the cell wall and to wash the material from the tissue, but the excessive use of these agents can cause protein denaturation [13]. In many cases, more than one of these classes is used for a higher level of promotion of decellularization [6,14].

Even though the method is widely used, problems remain concerning the best decellularization protocol and what type of cells to use [15]. The posterior recellularization of the graft still presents a challenge in the field. Various cell sources can be used to repopulate a decellularized scaffold, ranging from autologous somatic cells from the target tissue to stem cells in different stages of differentiation [16]. The use of autologous cells can avoid the problem of immune system rejection.

The subsequent transplantation of the created graft is fully dependent on the success of the previous steps, and transplantation in vivo of a fully functional and viable engineered organ through decellularization/recellularization is yet to be reported [17]. The attempts until now have not been able to secure graft survival for more than a few weeks [17] .

Good cell candidates for promoting scaffold recellularization are mesenchymal stromal/stem cells (MSCs). These cells have the potential for differentiating into hepatocyte-like cells [18] and endothelial cells [19] and can be obtained from various types of tissue from the patients themselves, thus eliminating the problem of immune rejection [20]. In this work, MSCs extracted from exfoliated deciduous teeth were used to promote recellularization.

MSCs from exfoliated deciduous teeth were used in association with polycaprolactone electrospun scaffolds to study the effect of flow rate and shear stress, adhesion time and seeding density under direct perfusion in a bioreactor [21]. MSC derivates of exfoliated deciduous teeth modulated early astrocyte response after spinal cord contusion [22]. Furthermore, it has been shown that cultivating MSCs with the use of a hollow fiber bioreactor can improve MSCs-derived hepatocyte-like cell maturation in vitro [23].

The recellularization of organs with cells can be achieved in essentially two ways. The first is to inject them directly on the parenchyma and the second is to introduce them via the vascular network; a combination of both is also possible. The advantage of using the second is that the cells are more evenly distributed throughout the tissue [24].

A good strategy that has been used in the recellularization process and subsequent cultivation of the inserted cells is the use of bioreactors. A bioreactor consists of an apparatus where biological or biochemical processes occur under monitored and controlled conditions. Such conditions can include the pH of the medium, the temperature, the pressure, nutrient supply and gas exchange [25]. This characteristic allows for high reproducibility and processing on an industrial scale of the products generated [26].

To be used in a cell cultivation system, a bioreactor needs to be able to promote adequate transportation of nutrients and oxygen as well as be able to efficiently remove metabolic waste. It is desirable that they are easy and quick to assemble and handle, enabling the production of their products quickly and efficiently. The material used for their manufacture must be free of toxicity and must be easily washable and sterilizable, thereby allowing for their reuse [26].

A perfusion system was chosen because it can use the native vascular structure, evenly distributing the solution used for decellularization and additionally promoting a balanced delivery of nutrients to the cells while removing metabolic dejects in the recellularization process. This is particularly important in liver grafts as oxygen delivery is crucial for hepatocytes survival [27].

In this work, the possibility of using an easy-to-assemble bioreactor to decellularize and recellularize a full liver graft with MSCs has been shown. This demonstrates the possibility of producing a bioreactor that meets the necessary specifications while generating low cost, thus facilitating its implementation in public health systems. The grafts maintained the structure and composition of the native liver and the cells were able to repopulate the scaffold and preserve its viability and activity.

## 2. Materials and Methods

### 2.1. System/Bioreactor Assembly

**Figure 1:**
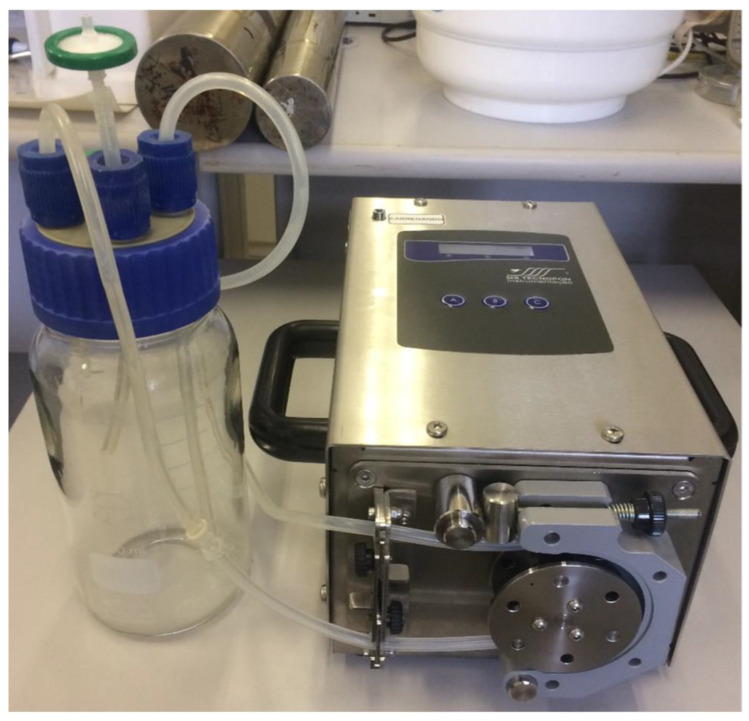
Bioreactor ready to use; on the left is the glass recipient used as organ chamber and on the right the pump used to create liquid flow. In the cover, three entries were used; the left one for liquid entrance, the middle one for air exchange (a filter was used to maintain sterility), and the right one for liquid exit.

The bioreactor was made with the assistance of the company Eva Scientific, which was hired for the purpose. A glass recipient with 1 L capacity was used as the organ chamber. The cover had four cylindrical entries, of which two were used as the entry and exit path for the liquid flow, one was used as the air flow with a 0.22 µm filter and the other remained closed. Silicone hoses with 6 mm external diameter and 3 mm internal diameter were used, and a Luer lock adapter was inserted in the entry way as a means of seeding the cells. The apparatus is easily cleaned, and its parts can be autoclaved for sterilization. A peristaltic pump was used to create the liquid flow (CDP-201-3—MS Tecnopon Equipamentos Especiais, Piracicaba, Brazil) (Figure 1).

### 2.2. Liver Harvest

Fresh livers were obtained from male Wistar rats and male Wistar Kyoto rats and washed with phosphate-buffered saline (PBS) containing 2 mM EDTA. A total of 10 livers were used in this study. The rats had the health status of conventionally monitored animals and were not genetically modified as knock-out or knock-in models. The portal vein was preserved to be used as the access for the perfusion. The organs were frozen at −80 °C. All methods were carried out in accordance with relevant guidelines and regulations and all experimental protocols were approved by the university’s ethics committee for animal experimentation (CEUA-UFRGS project number 32653). The organs were obtained from animals used in other projects that were already scheduled to be euthanized.

### 2.3. Isolation, Cultivation and Characterization of Mesenchymal Stromal/Stem Cells

The cells used in this work were isolated and characterized in other studies from our group, with MSCs isolated from human deciduous teeth pulp, following a protocol established in the laboratory [22]. All the methods were carried out in accordance with relevant guidelines and regulations and all experimental protocols were approved by the research ethics committee of the university (CEP-UFRGS) under CAAE 68383417.0.0000.5347. Informed consent was obtained from all the subjects or a parent and/or legal guardian.

The cells were cultivated in Dulbecco’s Modified Eagle’s Medium (DMEM, (Sigma-Aldrich, St. Louis, MI, USA) containing 2.5 g/L of Hepes (free-acid) (Sigma-Aldrich, St. Louis, MI, USA) supplemented with 10% bovine fetal serum (*v*/*v*) (BFS) (Gibco, Grand Island, NE, USA), 1% penicillin/streptomycin (*v*/*v*) (Gibco, Grand Island, NE, USA), amphotericin and 0.40 μg/mL gentamicin (Novafarma, Anápolis, Brazil). The culture was placed in a humid atmosphere of 5% CO_2_, at 37  °C. The culture medium was changed every three or four days.

The characterization occurred at the fifth passage and was performed by morphological analysis, differentiation assay in vitro (adipogenic, chondrogenic and osteogenic) and immunophenotypic analysis with flow cytometry (FACS Aria III BD, CA) with specific monoclonal antibodies (CD14, CD29, CD34, CD44, CD45, CD73, CD90, CD105 and CD184) (Pharmingen, Becton Dickinson, San Jose, CA, USA) [22].

### 2.4. Liver Decellularization

The frozen organs were thawed for 3 h under UV light. The perfusion occurred inside the bioreactor through the portal vein. To access the portal vein, a silicone urethral catheter fr 4 was used. The livers were perfused with distilled water for 24 h, then with 0.5% (*v*/*v*) sodium dodecyl sulfate (SDS) for 24 h and finally with distilled water for a further 24 h. The decellularized matrix was sterilized with a 0.1% peracetic acid (MedQuímica, Juiz de Fora, Brazil) solution in 4% ethanol (Sigma, St. Louis, MI, EUA) for 3 h and washed with PBS containing 1% of amphotericin, penicillin and streptomycin and 0.40 μg/mL of gentamicin for 24 h. The flow rate used for perfusion during decellularization was 5 mL/min for all the processes.

The decellularization was assessed macroscopically by the change of color in the organs and histologically by DNA, collagen and glycosaminoglycans quantifications, described in the text below.

### 2.5. Liver Recellularization

The decellularized matrices were perfused prior to cell seeding with DMEM containing 2.5 g/L of Hepes (free-acid) (Sigma-Aldrich, St. Louis, MI, EUA) supplemented with 10% bovine fetal serum (*v*/*v*) (BFS) (Gibco, Grand Island, USA), 1% penicillin/streptomycin (*v*/*v*), amphotericin and 0.40 μg/mL gentamicin for 24 h. The seeding was carried out in a multistep procedure, as described by Uygun et al. [9]. A total of 10^8^ undifferentiated MSCs cells were seeded and divided equally into four intervals with 10 min between each. At the end of the seeding, the perfusate was collected and the number of cells not retained in the liver was determined with a hemocytometer and trypan blue exclusion. The total number of cells remaining in the scaffold were estimated by subtracting the total number of cells seeded from the cells that were in the perfusate. The bioreactor was put inside a CO_2_ incubator and the recellularized graft was cultivated for seven days in a humid atmosphere of 5% CO_2_, at 37 °C. The culture medium was changed daily. For the liver recellularization and culture, the flow rate used was 5 mL/min. All the procedures were performed under sterile conditions.

### 2.6. DNA Quantification

The DNA was extracted with Trizol (Invitrogen), following the manufacturer’s protocol, and quantified by spectrophotometry. Tissue portions from the fresh and decellularized liver were homogenized and incubated, and 1 mL of Trizol reagent was added into the homogenate. The samples were then centrifuged for five min at 12,000× *g* at 4 °C. The supernatant was removed and 0.2 mL of chloroform (Neon Comercial, Suzano, Brazil) was added to the samples and incubated for three min. Following this, the samples were centrifuged for 15 min at 12,000× *g* at 4 °C. The aqueous phase was then discarded, and 0.3 mL of 100% ethanol (Sigma-Aldrich) was added to the remaining liquid and mixed by inverting the tube several times. The resulting mixture was incubated for three min and centrifuged for five min at 2000× *g* at 4 °C. The phenol-ethanol supernatant was discarded. The DNA mixture was resuspended in 1 mL of 0.1 M sodium citrate in 10% ethanol, incubated for 30 min and centrifuged for five min at 2000× *g* at 4 °C. This step was repeated three times. Following this, the remaining pellet was resuspended in 2 mL of 75% ethanol, incubated for 20 min and centrifuged for five min at 2000× *g* at 4 °C. The supernatant was discarded and the pellet was air-dried for 10 min. The dry DNA sample was resuspended in 0.6 mL of 8 mM NaOH and centrifuged for 10 min at 12,000× *g* at 4 °C to remove insoluble materials. The supernatant was transferred to a clean tube and the absorbance was measured at 260 nm and 280 nm (Nano Drop 2000—Thermo Fisher Scientific, Wilmington, CA, USA).

### 2.7. Collagen Quantification

Collagen was measured indirectly by the OH-proline assay [28]. Tissue portions from the fresh and decellularized liver were hydrolyzed with 6N HCl at 105 °C for 18 h. The HCl was evaporated in a vacuum desiccator. After evaporation, the samples were diluted in 50 μL of H_2_O, and 450 μL of a chloramine-T solution (0.056 M) was added to the samples and kept at room temperature for 25 min for oxidation. After this 25 min period, 500 μL of Ehrlich’s reagent was added (*p*-dimethylaminobenzaldehyde dissolved in methanol) and the samples were incubated for 20 min at 60 °C. A control curve was performed using a solution of collagen 2.000 ng/mL, serially diluted. The absorbance of the samples was measured in a spectrophotometer at a wavelength of 570 nm. The experiment was realized in three different livers in triplicate.

### 2.8. Glycosaminoglycans Quantification

Glycosaminoglycans were measured by the dimethylmethylene blue assay [29]. Tissue portions from the fresh and decellularized liver were homogenized in trypsin-EDTA solution and incubated for 18 h at 37 °C. A quantity of 200 μL of a blue solution of dimethylmethylene was added to 20 μL of the resulting homogenate. A control curve was established with a solution of chondroitin sulfate (500 μg/mL), serially diluted. The absorbance of the samples was measured in a spectrophotometer at a wavelength of 450 nm. The experiment was realized in three different livers in triplicate.

### 2.9. Histological Analysis

All the matrices, the fresh, decellularized, and recellularized, were fixed in 10% buffered formalin (pH 7.4) for 24 h, embedded in paraffin, and sliced to a 5 μm thickness. The slices were stained with hematoxylin & eosin (HE) and Masson’s trichrome. An optical microscope was used to visualize the slides.

### 2.10. Scanning Electron Microscopy (SEM)

For the ultrastructural analysis by SEM, the matrices were fixed in 4% paraformaldehyde for 24 h. The samples were frozen in liquid nitrogen and a scalpel was used to section them. The sections were placed on a carbon ribbon in microscopy stubs and metalized with gold. The decellularized matrix was evaluated with a scanning electron microscope (JSM-6060—JEOL, Tokyo, Japan).

### 2.11. Functional Analysis

At the time periods of one, three and seven days, the perfusate from the bioreactor culture with undifferentiated MSCs was extracted and levels of albumin, urea and lactate dehydrogenase (LDH) were measured by automated enzymatic colorimetric assays (Konelab 30i-Thermo Fisher Scientific, Oberhausen, Germany). The same cells used in the recellularization were cultivated in a six-well plate and used as the control for all the analyses and as a cell death control at each time point; Triton-X 10% was added to one of the wells. To preserve the comparability, a ratio of 10,000 cells/mL was used in the culture.

### 2.12. Statistical Analysis

For the DNA, collagen and glycosaminoglycans, the Student *t*-test was used. For the LDH, albumin and urea, two-way ANOVA was used followed by a Tukey HSD post hoc test. For statistical significance, a *p*-value of 0.05 was considered.

## 3. Results

### 3.1. System/Bioreactor Assembly

The system (Figure 1) was assembled in accordance with the specifications necessary for its use both for the decellularization process and recellularization process and as a bioreactor for cultivating the MSCs.

The peristaltic bomb has a digital display to choose the desired flow rate and can be changed on demand before and during the process. There is access to link it to a computer interface for more advanced programs if necessary. The silicon hoses can be changed after every process. The liquid flow could be created in a closed system.

The apparatus remained sterile after the material was autoclaved and handled under sterile conditions in a laminar flow hood. After being placed in cultivation conditions in a cell incubator with just the culture, the medium did not show microbial growth, as evidenced by the absence of turbidity and the negative growth of microorganisms in the blood and Sabouraud agar plates.

### 3.2. Liver Decellularization

**Figure 2:**
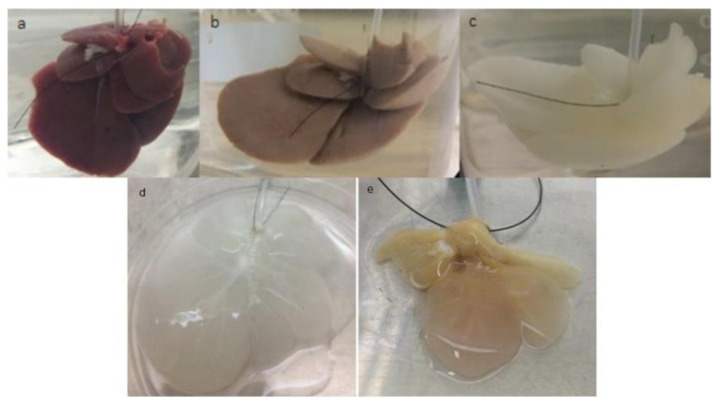
Evolution of decellularization and recellularization over time. Fresh liver placed in the system (**a**). Liver after 24 h perfusion with distilled H_2_O, showing a loss of coloration (**b**). Liver after infusion for 24 h with sodium dodecyl sulfate (SDS), showing a translucent coloration, indicating cell removal (**c**). Decellularized liver (**d**). Recellularized liver after seven days of cultivation in the bioreactor (**e**).

Figure 2a shows the appearance of a fresh liver placed in the system. For decellularization by perfusion, intact rat livers were placed, connected by the portal vein in the bioreactor. After 24 h with perfusion of distilled water, the organ showed a visual alteration in color indicated by the loss of pigmentation (Figure 2b), and after 48 h of decellularization with SDS, the organ lost all coloration, acquiring a white translucent color (Figure 2c).

**Figure 3:**
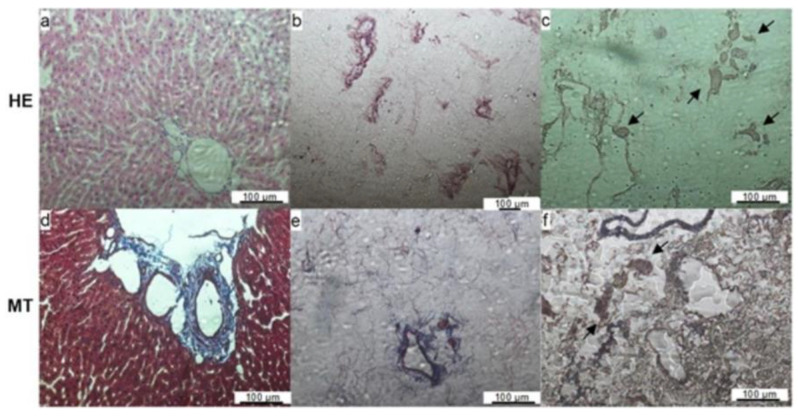
Histological sections of fresh (**a**,**d**), decellularized (**b**,**e**), and recellularized livers (**c**,**f**) after staining with hematoxylin & eosin (HE) or Masson ’s trichrome (MT). The absence of nuclei and maintenance of the extracellular matrix can be observed in the decellularized liver images. The presence of cells on the images of the recellularized liver (arrows) indicates that the cells were able to migrate from the vessels to the tissue. Scale bar represents 100 μm.

The histological coloration with hematoxylin and eosin of a fresh rat liver showed numerous nuclei and cytoplasmic components in the native tissue (Figure 3a). The HE showed an absence of cells in the decellularized matrix (Figure 3b), confirming the efficiency of the protocol. Collagen distributions were observed by Masson trichrome staining in both the fresh and the decellularized liver (Figure 3d,e, respectively).

**Figure 4:**
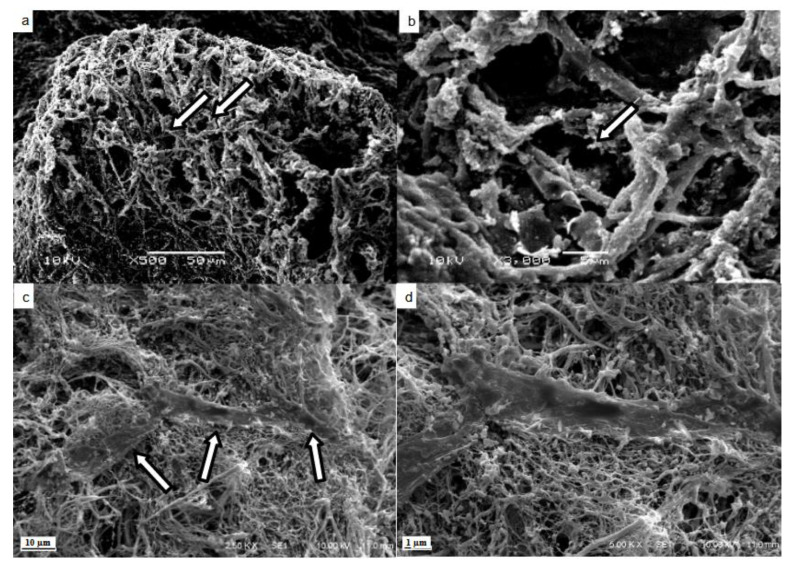
Scanning electron microscopy of decellularized and recellularized matrices. Maintenance of spaces (arrows) occupied by hepatocytes in the connective tissue tangle (**a**). Maintenance of fibers and extracellular matrix (arrow) in the parenchyma (**b**). Mesenchymal stromal/stem cells (arrows) attached to the matrix fibers (**c**). Mesenchymal stromal/stem cell attached to the matrix fibers amplified (**d**). Scale bar represents 50 μm (**a**), 5 μm (**b**), 10 μm (**c**) and 1 μm (**d**).

The SEM images of the decellularized matrix confirmed the efficiency of the protocol in removing cells and maintaining the fibers and ECM skeleton (Figure 4). The pictures show the empty spaces for the hepatocytes; the vessels were preserved with the procedure (Figure 4a,b).

**Figure 5:**
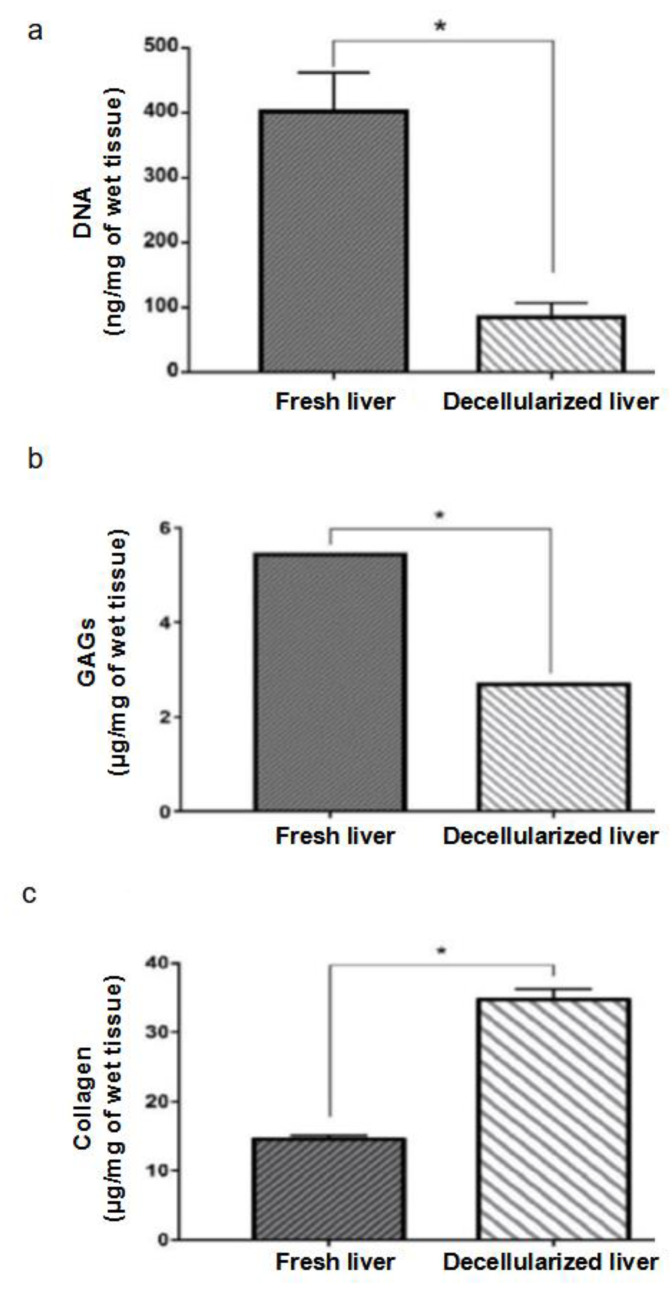
DNA quantification. Decellularized livers showed 84.8 ± 13.0 ng/mg DNA (*n* = 3), less than 100 ng/mg of wet tissue, which characterizes the absence of cells, in accordance with the literature. Fresh livers showed 401.6 ± 34.8 ng/mg DNA (*n* = 3). Values expressed as mean and standard deviation. * *p* < 0.05 by the Student *t*-test (**a**). Quantification of glycosaminoglycans (GAGs). Decellularized livers maintained approximately 50% of the amount of GAGs (2.69 ± 0.01 μg/mg, *n* = 3) found in the fresh liver (5.41 ± 0.01 μg/mg, *n* = 3). Values expressed as mean and standard deviation. * *p* < 0.05 by the Student *t*-test (**b**). Collagen quantification. Decellularized livers showed an increase in the amount of collagen per tissue mass (34.76 ± 0.87 μg/mg, *n* = 3) when compared to the fresh liver (14.58 ± 0.30 10 μg/mg, *n* = 3). Values expressed as mean and standard deviation. * *p* < 0.05 by the Student *t*-test (**c**).

The DNA quantity in the decellularized liver was significantly lower than 100 ng/mg of wet tissue (84.8 ± 13.0 ng/mg, *n* = 3), while in the fresh liver it was 401.6 ± 34.8 ng/mg, *n* = 3, indicating that the protocol was efficient in removing nuclear material (Figure 5a). The glycosaminoglycans were preserved in 50% (2.69 ± 0.01 µg/mg, *n* = 4) of their native quantity (5.41 ± 0.01 µg/mg, *n* = 3), showing that the processes can maintain the structural content while removing cellular material (Figure 5b). The collagen measured showed an increase in its quantity per mg of wet tissue from 14.58 ± 0.30 µg/mg, *n* = 3, to 34.76 ± 0.87 µg/mg, *n* = 3 (Figure 5c), further indicating that the processes can maintain the structural content while removing cellular material.

### 3.3. Liver Recellularization and Functional Analysis

After recellularization, the number of undifferentiated MSCs counted that were not retained in the scaffold was 3% of the total, indicating that 97% of the cells were retained in the scaffold.

Figure 2d shows the macroscopic appearance of a translucent decellularized liver while Figure 2e shows the increase in the coloration, going from translucent to a light shade of brown in the recellularized liver with MCSs, cultivated in the bioreactor for seven days.

The histological evaluation with HE showed some cells present close to the vessel space in the recellularized matrix (Figure 3c). Masson’s trichrome staining showed the maintenance of ECM components such as the collagen web in the recellularized matrices (Figure 3f). The SEM images show MCSs attached to the matrix fibers, confirming that the matrix was recellularized by the protocol (Figure 4c,d).

**Figure 6:**
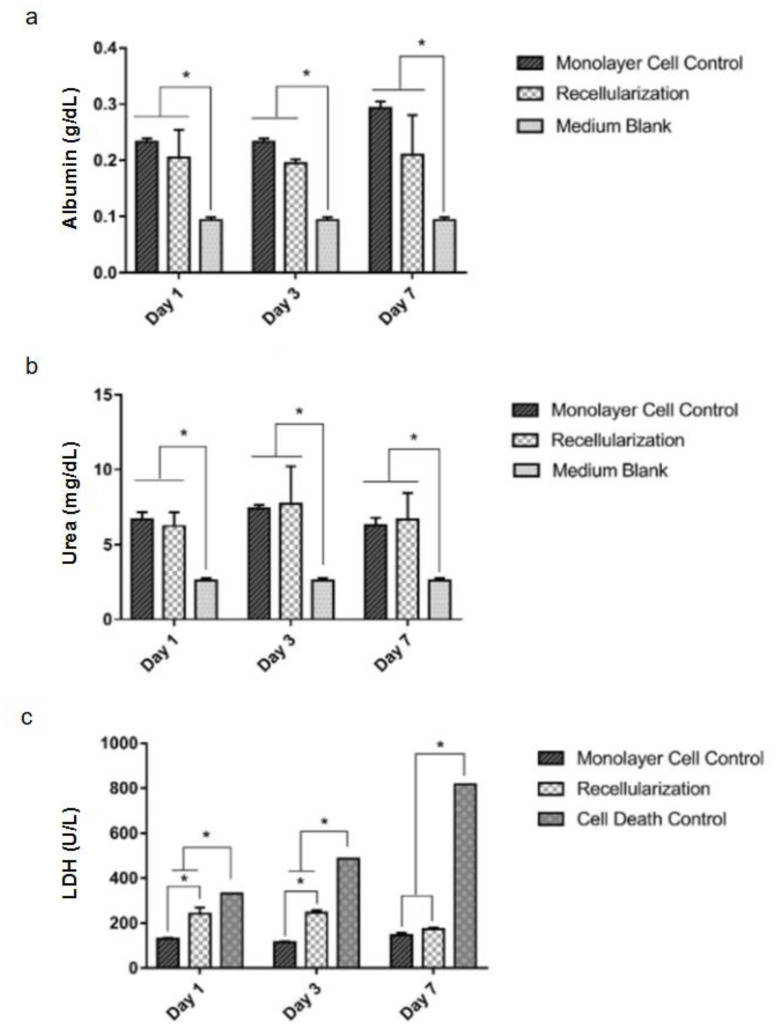
Results of functional analyses of recellularized livers. Albumin (g/dL) (**a**). Urea (mg/dL) (**b**). Lactate dehydrogenase (LDH in units per liter, U/L) (**c**). Medium blank represents the medium used for the cell culture to remove color interference. Data presented as mean and standard deviation, *n* = 3. * *p* < 0.05 by two-way ANOVA followed by Tukey HSD post hoc test.

Statistically, similar amounts of albumin (Figure 6a) and urea (Figure 6b) were detected in the 6-well plate cell culture (monolayer control) and the recellularized liver cell culture, indicating that there is no differentiation into hepatocyte-like cells after a week. The culture medium without cells (blank) presented statistically significant lower albumin and urea contents in the time points tested (Figure 6a,b, respectively). The LDH quantity was slightly higher than the control at one and three days but showed no difference at seven days (Figure 6c). In addition, for the cell death control, a detergent lyse was added to the cells, resulting in a higher LDH content than the monolayer control and the recellularized sample.

## 4. Discussion

These results present the possibility of using common and easy-to-assemble materials to produce a bioreactor that can be used for biological processes. The use of systems, such as that produced in this work, is recommended in decellularization and recellularization protocols because of the mass delivery control and constant removal of the metabolic dejects [26]. The technology of liver decellularization by perfusion already allows the production of acellular scaffolds with the vascular tree well preserved and with clinical application potential [28,29]. In a recent study by Wang and colleagues (2023), the rats livers were decellularized by perfusion with 10% SDS at 2 mL/min in a similar process to the one used in the present work. For the assembly of a bioengineered construct, the authors recellularized the scaffolds with hepatocytes. The rats with acute liver failure transplanted with the tissue-engineered liver presented longer survival time than hepatectomy rats [30]. Our investigation differs from this work due to the fact that aspects of the production of the machine used for decellularization and recellularization were highlighted.

In recent years, the acellular extracellular decellularized matrices of livers have been used to produce hydrogels. This material is then processed (undone in relation to the shape of the organ) and mixed with cells, to fabricate bioinks that are customary in bioprinting [31]. Thus, the protocol for decellularization described in the present paper can be used to manufacture optimized biomaterials. The decellularized matrix can also be applied to fabricate other forms of scaffolds. For example, in the study of Mahbud and colleagues (2023), the decellularized matrix of livers was freeze-dried, ground, and combined with thrombin, oxidized cellulose, and chitosan for hemostasis and wound-healing in rat liver hemorrhage [32]. As previously mentioned, the decellularization protocol established in the present research may be employed for making different types of matrices for a variety of tissue-repair applications.

Furthermore, bioreactors are useful tools in tissue engineering, when compared with other culture techniques, because they can reduce limitations that occur in mass transport processes in vitro. This happens because the system creates a dynamic cultivating environment that efficiently mimics the natural environment in the original tissue. Bioreactors can also optimize the specific process, such as cell adhesion, expansion and differentiation [33]. It additionally permits the control of parameters such as flow rate, increasing the reproducibility and eliminating the variability in the system, when compared to other perfusion protocols [25]. It is important to note that although the dynamic system in bioreactors better mimics the physiological environment in vivo [34], shear stress needs to be optimized in the process of de- and recellularization. For example, the shear stress provided by the dynamic culture systems can stimulate extracellular matrix synthesis [35] and induce cell migration [36]. On the other hand, extreme mechanical stress has been shown to promote cell necrosis [37]. In the case of perfusion bioreactors, the flow provides higher nutrient transport, higher cell viability, as well as uniformity of cell distribution [33,38,39].

The computational estimation studies of shear stress are complex and involve interactions between the matrices, cells, and the culture system of tissue development in vitro. The models to predict tissue development have limitations and are challenging [40]. In a study of computational fluid dynamics focusing on the effect of the flow rate, the high flow (20, 40, and 60 mL/min) increased the shear stress on the scaffold structure. In contrast, low levels of stress were found in the use of low flow rates (1.5, 3.0, and 4.5 mL/min) [41]. A paper from 2012 showed the computer simulation of a bioreactor for the bioartificial liver system with a flow rate of 1, 2, 5 and 10 mL/min that presented lower shear stress than the critical value (33 mPa). This work of Xia and colleagues found a value of 1.9 mPa with a flow of 5 mL/min, the same flow used in the present study. Additionally, the secretion of albumin from hepatocytes in the perfusion culture with 5 mL/min had a huge increase on days two and three and reduced from day four [42]. Hussein and colleagues (2016) added heparin-gelatin to the decellularized porcine livers. Subsequently, the researchers recellularized with co-cultures of hepatocellular carcinoma (HepG2) and endothelial cells and kept it in the bioreactor with a flow rate adjusted to 5–8 mL/min for 10 days. The results indicated no shear stress on HepG2 cells in parenchymal and suitable cell function in vitro and in vivo [43].

In addition, the apparatus is easily cleaned and its parts can be autoclaved for sterilization. This greatly reduces the cost of applying the procedure on a large scale, making this viable for application in public health systems. Furthermore, the apparatus is intuitive and has a low learning curve, reducing the time needed for training health workers to use it.

The liver and matrix composition and tridimensional microstructure were maintained after the process, as shown in the SEM analysis. The empty spaces where the hepatocytes used to be can be seen in the midst of the ECM. The decellularized scaffold showed a DNA content lower than 100 ng/mg of wet tissue; this value represents a threshold for confirming cell removal [44]. Histological analysis corroborates these results with no nucleus colored in the decellularized matrix.

The collagen quantity in the scaffold was significantly higher than in the fresh liver because the loss of cells also represents a loss of mass. This result indicates that most of the remaining scaffold mass is composed of collagen [45]. These results are compatible with other perfusion protocols that show an increase of approximately two times in wet tissue collagen content when using animal organs [14,46]. The retention of the quantity and the maintenance of the integrity of collagen are important for preserving the mechanical properties of the scaffolds [14].

The amount of glycosaminoglycans kept in the scaffold was approximately 50% of the total in the fresh liver. This decrease is because GAGs are also part of the cell membrane, which was removed during the decellularization process. These substances can aid in cell differentiation and are responsible for the storage of various cellular communication proteins, which favor proliferation, maintaining them in the scaffold at appropriate levels. This can be useful for future applications, such as testing the differentiation of stromal/stem cells using the scaffold as a guide [47,48].

As human hepatocytes have poor proliferative potential in vitro, the use of stem cells to repopulate the decellularized liver is a recommended approach because they have a high capacity for expansion and can differentiate into a variety of tissue types, including hepatocyte-like cells [18]. MSCs have an advantage over other types of stem cells because there is no ethical issue regarding their use, and they do not have tumorigenic problems when compared with embryonic stem cells or induced pluripotent stem cells [49]. Furthermore, it is known that the transplantation of MSCs or MSC-derived hepatocyte-like cells in patients suffering from liver damage can help improve liver function [49]. This, coupled with the fact that hepatocytes are difficult to obtain, makes MSCs good alternatives for use in liver bioengineering.

To show the aptitude of the scaffold as a cell container, it was successfully repopulated with MSCs. The chosen number of cells, 10^8^, represents 10% of the liver cell population in a rat [50], which is the minimum necessary to restore the liver function in a graft. Likewise, the absence of cells during decellularization produces a liver without color. Figure 2e shows a homogeneous coloration provided by the liver recellularization and culture for seven days in the bioreactor.

The cells after seven days were alive and had metabolic activity compatible with mesenchymal stromal/stem cells, as shown by the LDH, albumin and urea quantities.

The LDH analysis indicated that the cells showed a tendency to die in the first three days of cultivation in the bioreactor; but on the seventh day, the LDH levels in the recellularized graft were not statistically different from the cells grown on the plate. These data suggest that on the seventh day there was no further cell death. The initial cell death probably occurs because of the shear stress caused by the perfusion process [33]. As time passes, the cells adjust to the new environment.

The results show that there is no difference in the levels of albumin and urea from MSCs cultivated on the culture plate and the recellularized scaffold. This minimal albumin production by MSCs was detected previously [51]. The low detection of albumin and urea was an expected result because the process of differentiation in hepatocyte-like cells requires supplying them with different factors, such as epithelial growth factor, hepatic growth factor and oncostatin to induce it [49,51,52]. In this process, it was shown that the ECM is proven to be a facilitator of cell differentiation [53].

This work demonstrates the viability of the system for promoting decellularization and recellularization. With the bioreactor able to maintain the cell culture, the next step is to induce the differentiation over a prolonged cultivation time. Furthermore, the perfusion method guarantees a better distribution of the substances in the scaffold, facilitating its differentiation [14].

With this work, it has been possible to establish a perfusion system for decellularization and a bioreactor system to use for the subsequent recellularization method. The designed apparatus is easy to use and can support the cultivation of mesenchymal stromal/stem cells in a liver scaffold.

## Data Availability

The data that support the findings of this study are available from the first author upon reasonable request.
